# T-cell branched glycosylation as a mediator of colitis-associated colorectal cancer progression: a potential new risk biomarker in inflammatory bowel disease

**DOI:** 10.1093/ecco-jcc/jjaf043

**Published:** 2025-03-15

**Authors:** Eduarda Leite-Gomes, Mariana C Silva, Ana M Dias, Ângela Fernandes, Guilherme Faria, Rafaela Nogueira, Beatriz Santos-Pereira, Henrique Fernandes-Mendes, Catarina M Azevedo, Joana Raposo, Julian López Portero, Tania de Alda Catalá, Carlos Taxonera, Paula Lago, Maria J Fernandez-Aceñero, Isadora Rosa, Ricardo Marcos-Pinto, Salomé S Pinho

**Affiliations:** Institute for Research and Innovation in Health (i3S), University of Porto, Porto, Portugal; School of Medicine and Biomedical Sciences (ICBAS), University of Porto, Porto, Portugal; Institute for Research and Innovation in Health (i3S), University of Porto, Porto, Portugal; School of Medicine and Biomedical Sciences (ICBAS), University of Porto, Porto, Portugal; Institute for Research and Innovation in Health (i3S), University of Porto, Porto, Portugal; Institute for Research and Innovation in Health (i3S), University of Porto, Porto, Portugal; Institute for Research and Innovation in Health (i3S), University of Porto, Porto, Portugal; School of Medicine and Biomedical Sciences (ICBAS), University of Porto, Porto, Portugal; Institute for Research and Innovation in Health (i3S), University of Porto, Porto, Portugal; Institute for Research and Innovation in Health (i3S), University of Porto, Porto, Portugal; Faculty of Medicine, University of Porto, Porto, Portugal; Department of Gastroenterology, Centro Hospitalar Universitário de Santo António, Porto, Portugal; Institute for Research and Innovation in Health (i3S), University of Porto, Porto, Portugal; School of Medicine and Biomedical Sciences (ICBAS), University of Porto, Porto, Portugal; Department of Surgical Pathology, Centro Hospitalar Universitário de Santo António, Porto, Portugal; Department of Surgical Pathology, Hospital Clínico San Carlos, Madrid, Spain; Department of Surgical Pathology, Hospital Clínico San Carlos, Madrid, Spain; Department of Gastroenterology, Hospital Clínico San Carlos, Madrid, Spain; Department of Gastroenterology, Centro Hospitalar Universitário de Santo António, Porto, Portugal; Department of Surgical Pathology, Hospital Clínico San Carlos, Madrid, Spain; Department of Gastroenterology, Instituto Português de Oncologia de Lisboa Francisco Gentil, Lisboa, Portugal; Institute for Research and Innovation in Health (i3S), University of Porto, Porto, Portugal; Department of Gastroenterology, Centro Hospitalar Universitário de Santo António, Porto, Portugal; Institute for Research and Innovation in Health (i3S), University of Porto, Porto, Portugal; School of Medicine and Biomedical Sciences (ICBAS), University of Porto, Porto, Portugal; Faculty of Medicine, University of Porto, Porto, Portugal

**Keywords:** colitis-associated colorectal cancer, T cells, glycans

## Abstract

**Background and Aims:**

Inflammatory bowel disease (IBD) is a chronic inflammatory disorder of the gastrointestinal tract, established as a risk factor for colorectal cancer (CRC) development. Long-standing inflammation appears to play a central role in colitis-associated colorectal cancer (CAC). However, the molecular mechanism underlying CAC progression is still elusive. Previous evidence showed that levels of branched glycosylation regulate T-cell-mediated immune response associated with IBD severity. Here, we revealed that colonic T cells from IBD patients are dynamically regulated by branched* N*-glycosylation and associated with the risk of CAC development.

**Methods:**

We performed *in silico* analysis for glycome and immune profile of a publicly available human dataset of CAC patients. Additionally, in a well-characterized cohort of CAC patients, we evaluated the *N*-glycosylation profile of infiltrated colonic immune cells at different stages of carcinogenesis (colitis, dysplasia and cancer). *In vivo* studies were conducted in *Mgat5* KO mice, using AOM/DSS model to induce CAC. Tumor development and colonic T cells glycoprofile were characterized during CAC development.

**Results:**

The combined analysis of human IBD and CAC clinical samples, together with glycoengineered mouse model susceptible to CAC, revealed a gradual and dynamic increase of branched *N*-glycans in T cells from colitis to dysplasia and cancer. This glycosylation switch was shown to impose inhibitory properties in T cells, precluding an effective antitumor immune response. Mechanistically, we demonstrated that the deletion of branched *N*-glycans in *Mgat5* knockout mice led to CAC suppression due to increased infiltration of CD8^+^and γδ T cells, contributing to an effective antitumor immune response. From the clinical standpoint, we demonstrated that branched *N*-glycosylation levels detected in inflamed lesions from IBD patients predicted CAC progression with a sensitivity of 83.3% and specificity of 67.9% when assessed together with age at diagnosis.

**Conclusions:**

Overall, we here disclosed a new mechanism underlying CAC development, identifying a potential clinical biomarker plausible to improve the efficacy of cancer surveillance programs through the early identification of high-risk IBD patients, for preventive clinical and therapeutic strategies.

## Graphical Abstract

**Graphical Abstract:**
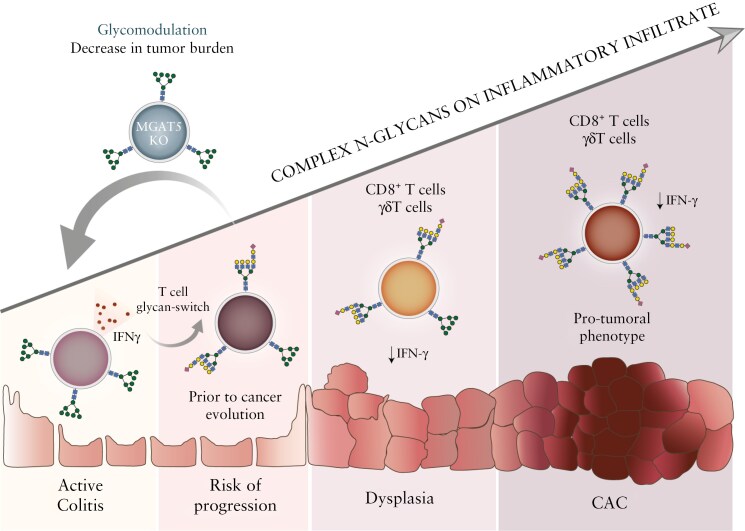

## 1. Introduction

Colitis-associated colorectal cancer (CAC) is a major complication of inflammatory bowel disease (IBD), remaining an important clinical challenge in terms of diagnosis and prognosis.^1^ In fact, patients with IBD present an increased risk for developing colorectal cancer (CRC), with earlier studies showing a 20- to 30-fold higher risk when compared to the general population.^2^ This risk increases with the duration of the disease (a cancer risk of 2% by 10 years, 8% by 20 years, and 18% by 30 years).^3^ It was also demonstrated that an average of 1.6% of patients with ulcerative colitis (UC) was diagnosed with CRC during 14 years of follow-up.^4^ Further studies showed that the adjusted CRC risk for all IBD patients is currently 2.2, but it can reach 7.0 for long-duration extensive colitis.^5,6^ Additionally, patients with UC-low-grade dysplasia (LGD) display an annual incidence of progression to cancer of 0.8%.^7^ Other risk factors include the anatomic extent of colitis, male sex, family history of CRC, the presence of colonic strictures or a tubular colon, a previous dysplasia diagnosis, and primary sclerosing cholangitis (PSC).^8,9^ Although evidence shows that the severity, persistence, and extension of the chronic inflammation of the colon are important factors associated with the development of CAC,^10^ the precise underlying molecular mechanism of inflammation-driven colon carcinogenesis is far from being fully elucidated, and thereby, there are no accurate and reliable prognostic biomarkers to stratify the risk for CAC progression and to early identify who will develop CRC. Moreover, the efficacy of cancer screening and diagnosis remains poor, as the current endoscopic surveillance for early detection of CAC is fraught with challenges and lack of consensus. Furthermore, CAC patients have worse 5-year survival rates in individuals below 50 years old when compared to sporadic CRC.^11^

In this study, we have investigated whether *N*-glycosylation alterations of colonic mucosa, specifically at the surface of lamina propria T cells, constitute a switching mechanism toward malignancy and thus, a putative prognostic biomarker to detect early preneoplastic alterations in IBD patients. In fact, glycosylation is one of the most important post-translational modifications, consisting of the addition of carbohydrate structures (glycans) to proteins and lipids of essentially all cells by specific glycosyltransferases and glycosidases enzymes.^12^ Changes in glycosylation are a hallmark of malignant transformation.^13^ Previous evidence from others and us have been revealing the key role that different glycans play in cancer and IBD,^14–21^ highlighting the relevance of branched *N*-glycans as master regulators of the inflammatory response in the context of IBD and other immune-mediated diseases.^16,22–26^ In fact, T-cell glycome has been demonstrated to tightly regulate T-cell activity and function.^24,27,28^ In IBD, we have previously demonstrated that UC patients exhibit a downregulation of the *MGAT5* glycogene and consequently a decrease in *N*-acetylglucosaminyltransferase V (GnT-V)-mediated branched *N*-glycans in intestinal T cells.^15^ This deficiency in complex branched *N*-glycans on mucosal T lymphocytes was associated with a hyperimmune response and intestinal inflammation as well as with disease severity.^15,16,29^ Conversely, in the context of gastrointestinal cancer, we have also demonstrated that *MGAT5* glycogene expression is upregulated, leading to the promotion of GnT-V-mediated branched *N*-glycan structures in cancer epithelial cells. This dysregulation promotes a pro-invasive and pro-metastatic behavior of tumor cells, further correlated with poor prognosis of cancer patients^17–19,30^ and shown to impact tumor immunoediting.^17^ However, whether this differential expression of branched *N*-glycans in IBD (downregulated in T cells) and CRC (upregulated in the cancer cells) constitutes a novel switching mechanism underlying malignant transformation and CAC development remains a key unanswered question. In this study, we demonstrated the dynamic regulatory properties of branched *N*-glycosylation in T-cell-mediated immune response during the transition from inflammation to dysplasia and colon cancer. The combination of human data from long-standing IBD and CAC patients together with the use of glycoengineered mice subjected to azoxymethane (AOM)/dextran sulfate sodium (DSS) for CAC induction revealed that intralesional T cells are dynamically regulated by branched *N*-glycosylation. This glycoprofile impacts T-cell activity and defines the risk and susceptibility to colon cancer progression. Importantly, from the clinical standpoint, we further demonstrated that levels of branched *N*-glycosylation in inflammatory infiltrates, detected in inflamed lesions of IBD patients, had the capacity to predict CAC progression with a sensitivity of 83.3% and specificity of 67.9% (area under the curve [AUC] of 0.827) when assessed together with age at diagnosis, which pinpoints a putative novel risk biomarker with potential to be included in the cancer risk algorithm of IBD patients.

## 2. Materials and methods

### 2.1. Bioinformatic data analysis

*In silico* analyses were performed in RStudio and pertained to datasets of human CAC^31^ and the AOM/DSS CAC mouse model.^32^ In both instances, raw CEL files were obtained through the Gene Expression Omnibus (GSE37283^31^ and GSE31106,^32^ respectively) and processed as described by Klaus and Reisenhauer.^33^ Briefly, CEL files were merged into an ExpressionSet object and quality control proceeded through adjustment of probe intensities relative to background noise, array normalization, and calculating the relative logarithmic expression of each probe across all samples. Subsequently, probes with low intensity were filtered and multiple mappings between probes and genes were removed—probe redundancy was resolved by choosing the probe with the highest measured intensity. This yielded final expression count matrices with log_2_ normalized probe intensity values. For the dataset obtained from Pekow et al., this produced a matrix of 27 800 genes across 16 samples (11 samples with CAC and 5 samples from healthy colon). One sample was removed after principal component analysis (PCA) since it revealed substantial variation in global gene expression relative to all other samples. For the dataset from Tang et al., the final expression matrix consisted of 20 838 genes across 18 samples.

Differential gene expression was performed on log_2_ normalized probe intensities with the limma package. Fold changes were computed with respect to either the normal or the inflamed colonic mucosa for all subsequent stages in the AOM/DSS dataset and between all conditions in the human CAC dataset. In both cases, *P*-values were adjusted for multiple comparisons based on the false discovery rate. To produce the corresponding volcano plots, detected glycosylation-related genes and their associated fold change and adjusted *P*-values were extracted and plotted with the EnhancedVolcano package. In addition, heatmaps were generated using the pheatmap package with Manhattan distances between *Z*-scored expression values and complete clustering between computed distances. For clustering analysis, the associated scores of glycogenes modules were calculated using the mean *Z*-scored expression values of the following sets/modules of genes: for *N*-glycan branching, MGAT1, MAN2A1, MAN2B1, MGAT2, MGAT3 and MGAT5; for sialylation, ST3GAL1 and ST6GAL1; for fucosylation, FUT2 and FUT8. To evaluate the concordance between the clustering, the Adjusted Rand Index (ARI) was computed, comparing the predicted label and the true label upon partitioning of 2 separate clusters. For the AOM/DSS dataset, dotplots of differential gene expression statistics were produced with the ggplot2 package.

PCA was performed for global gene expression and for selected genes referring to the *N*-glycosylation biosynthesis pathway. Principal component values were computed with the prcomp function of the stats package and plotted with the factoextra package, with concentration ellipses indicating confidence in the grouping of observations belonging to the same category.

Correlations between selected glycogenes and immune-related genes (markers for T cells, B cells, macrophages, and dendritic cells) were performed by calculating the Pearson correlation coefficient and associated *P*-values corrected (corrected for false discovery rate) using the psych package. Associated heatmaps were generated with the ggplot2 package. Correlation analysis was also performed using the clustering of glycogenes (complex branched *N*-glycans, sialylation, and fucosylation).

### 2.2. Human cohort

Bioinformatics analysis was performed based on a publicly available dataset,^31^ including patients with CAC lesions, in which PSC was not included. Samples include LGD, high-grade dysplasia (HGD), and adenocarcinoma. Two lesions were derived from the ascending colon, 3 from the transverse colon, 1 from the descending colon,; 3 from the sigmoid colon, and 2 from the rectum. UC patients with neoplasia had an average of 46.9 years old with an average of 13.5 years of disease duration and all samples were obtained from males. Healthy samples were also collected from males who had an average age of 46 years old. Additional information can be found in the original publication.^31^ Complementary, formalin-fixed paraffin-embedded (FFPE) sections (*n* = 190) of colonic mucosa were obtained from a cohort of IBD patients from *Centro Hospitalar Universitário de Santo António* (CHUdSA), Porto, Portugal (*n* = 155 FFPE; *n *= 31 patients) and from *Hospital Clínico San Carlos* (HCSC), Madrid, Spain (*n* = 35 FFPE; *n* = 18 patients). All IBD patients were included in the risk category for surveillance of CRC according to the European Crohn’s and Colitis Organization (ECCO) guidelines.^9^ These samples were organized in 2 different classifications regarding histopathological evaluation at the moment of the biopsy (remission/non-inflamed [*n* = 25], inflamed [*n* = 124], dysplasia [*n* = 34], or CAC [*n* = 7]) or regarding risk of CAC development according to the ECCO guidelines (low risk [*n* = 37], intermediate risk [*n* = 50], and high risk [*n* = 62]). Dysplasia was evaluated by expert Gastrointestinal Pathologists from the Pathology departments of the Hospitals (Porto, Madrid, and Lisbon). Dysplasia was classified as derived from IBD whenever it was detected in colonic segments known to be affected by IBD (endoscopic evidence of inflammation concurrently or in previous exams). Dysplasia associated with inflammation was detected in normal-appearing mucosa, in superficial flat lesions (Paris 0-IIa), or in elevated lesions (Paris 0-Is) with irregular/indefinite limits. Polyps or flat lesions with dysplasia that arise in areas with no documented history of inflammation were considered sporadic adenomas. Patients’ information is detailed in Tables S1 and S2. Colonic fresh biopsies from patients with risk for CRC development were collected at the *Instituto Português de Oncologia de Lisboa Francisco Gentil* (IPO), Lisbon, Portugal, and CHUdSA (*n* = 5; 2 biopsies in dysplasia and 3 biopsies under inflammation). Provided patients’ material and clinical information were used to perform lectin- and immunohistochemistry (IHC), flow cytometry analysis, as well as perform statistical analysis. The present study was approved by the ethics committee of the CHUdSA, HCSC, and IPO.

### 2.3. Animal model

All mouse procedures were approved by the Institute for Research and Innovation in Health (i3S) animal ethics committee for animal experimentation under Portuguese regulations (DGAV license number 009268/2022-06-02). Mice were housed at the Association for Assessment and Accreditation of Laboratorial Animal Care-accredited i3S animal facility in a temperature-controlled (20–24 °C) room maintained at a humidity of 45%–55% under a 12-hour light/12-hour dark period. C57BL/6 wild-type (WT) and *Mgat5* knockout (KO) mice (established and provided by Prof. Michael Pierce, CCRC, Georgia University, Athens, GA) with 8–10 weeks old were used for the induction of AOM/DSS CAC mouse model. This chemical-induced mouse model consists of the treatment with the genotoxic agent AOM combined with the repeated administration of DSS.^34^ Two independent experiments were performed using WT and *Mgat5* KO mice. 8–10 mg/kg of AOM (Sigma-Aldrich) were injected via intraperitoneal and DSS treatment started up to 7 days after AOM injection. Oral DSS (36.000–50.000 Da; MP Biomedicals) was carried in 3 cycles of 7 days (for the first cycle with 2% DSS) or 5 days (for the last 2 cycles with 1.5% DSS), with a recuperation period of 2 weeks after each cycle. Mice were monitored 3–5 times per week; rectal bleeding, stool consistency, and mice weight were assessed for disease activity index evaluation. To detect CAC development in different stages (colitis, dysplasia, or carcinoma), 4 different timepoints for euthanasia were considered across the disease course. The endpoint was determined by the presence of big lesions identified in colonoscopies or after 20 weeks from AOM injection. Mice were euthanized with lethal doses of isoflurane or CO_2_ and the colon was collected. Upon opening the colon, the entire luminal surface was observed, and the lesion size was measured at the last time point. Distal colon from AOM/DSS group was bisected longitudinally in 3 equal portions. One portion was stored in *RNAlater* stabilization solution (Sigma-Aldrich) for further transcriptome analysis and the other for flow cytometry analysis. The third portion of the colon was fixed in 4% formaldehyde (Enzymatic) for further paraffin embedding, processing, and slicing for hematoxylin and eosin (H&E) staining and lectin histochemistry.

### 2.4. Hematoxylin and eosin staining

Sections from the colonic tissue of AOM/DSS mice model were stained with H&E, in order to analyze histologically the morphology and stage of the lesions. Slides were stained with hematoxylin (Richard-Allan Scientific Modified Mayer’s Hematoxylin, Thermo Scientific), washed with water, and embedded in 1% ammonia water to differentiate the nuclei. The slides were washed again with water and ethanol 95% and then embedded in eosin (Eosin-Y, Thermo Scientific). Histological evaluation of the mice samples was performed by the pathologist and samples were regrouped according to the histologic classification referred to above (colitis, dysplasia, or carcinoma).

### 2.5. Lectin- and immunohistochemistry of L-PHA and CD3

For the IHC assays, FFPE colon tissue sections (4 µm) were stained for CD3 and Phaseolus vulgaris Leucoagglutinin (L-PHA), a lectin that recognizes β1,6-GlcNAc branched *N*-glycans. For L-PHA staining, sections were deparaffinized in xylene and rehydrated with successively lower concentrations of ethanol. After endogenous peroxidase blockage (3% H_2_O_2_ in methanol for 10 minutes), slides were blocked with BSA 10% for 30 minutes. Afterward, samples were incubated with biotinylated L-PHA (B-1115, Vector) in PBS 1 × (1:1000) for 1 hour. Then, the slides were washed 3 times with PBS 1x, followed by incubation with Vectastain ABC kit (Vectastain® ABC Kit, Vector Laboratories) for 30 minutes, 1:100 in PBS 1x. Next, color was developed after incubation with 3,3′-diaminobenzidine (DAB, K3468, Dako) for approximately 3 minutes in the dark, followed by counterstain with hematoxylin. Lastly, sections were dehydrated, preserved in a mounting medium (Entellan new, Sigma-Aldrich), and images recorded in a Brightfield Microscope (Leica DM2000 LED). The levels of expression of branched *N*-glycans on the intestinal infiltrate were evaluated by 3 independent observers and scored using a standard semi-quantitative method as follows: 0%–25%, 25%–50%, 50%–75%, and more than 75% of positive L-PHA reactivity. A receiver-operating characteristic (ROC) analysis was done and the cutoff of 50% was determined using SPSS software.

For CD3 staining, tissue sections were immunostained according to optimized protocols. The downstream IHC protocol for CD3 (Rabbit IgG anti-human CD3, clone SP7, RM-9107-S0, Epredia, 1:50) was performed on a fully automated Discovery ULTRA Platform (Ventana) using UltraView Universal DAB Detection Kit (Roche).

### 2.6. Immunofluorescence

For the immunofluorescence (IF) assay, FFPE colon tissue sections (4 µm) were stained for CD3 and L-PHA. Sections were deparaffinized, rehydrated, and submitted to antigen retrieval using EnVision FLEX Target Retrieval Solution, High pH (Dako Omnis) for 40 minutes in a steam cooking machine (Ufesa). Samples were incubated for 10 minutes in 3% H_2_O_2_ in PBS, blocked with goat normal serum (X0907, Dako) in bovine serum albumin (BSA) 10% (1:5) for 20 minutes, followed by CD3 staining (Rabbit IgG anti-human CD3, clone SP7, RM-9107-S0, Epredia, 1:50 in the blocking solution), O.N at 4 °C. Afterward, samples were washed and incubated with Goat anti-Rabbit IgG (H + L) Alexa Fluor 594 (Invitrogen, 1:800, in the blocking solution). Then, the slides were washed with PBS, followed by incubation with biotinylated L-PHA (B-1115, Vector, 1:500 in PBS 1x) for 1 hour. Samples were washed and incubated with Streptavidin Alexa Fluor 488 Conjugate (Invitrogen, 1:1000 in PBS 1x) for 45 minutes. Next samples were washed and incubated with DAPI (Sigma-Aldrich D9564, 1:50 000) for 5 minutes and sections were preserved in Vectashield antifade mounting medium (Vector Laboratories). Digitalized whole slides were obtained using Phenoimager HT 2.0 (Akoya Biosciences) and analyzed using the QuPath 0.5.1 and the image J Software.

### 2.7. Isolation of lamina propria immune cells

Distal colon from WT and *Mgat5* KO mice were collected at different timepoints (colitis, dysplasia, or carcinoma) and fresh colon biopsies were collected from patients during colonoscopy. Samples were manually minced and digested in a dissociation medium (RPMI 1640 GlutaMAX medium [Gibco] with collagenase IV (0.9 mg/mL; Sigma-Aldrich) supplemented with 10% FBS, 100 U/mL penicillin/streptomycin, 1 mmol/L CaCl_2_, and 1 mmol/L MgCl_2_) for 45 minutes with agitation at 37 °C. For human samples, flow cytometry staining was performed after the enzymatic digestion. For mice samples, after enzymatic digestion, immune cells were isolated with LymphoPrep solution, according to the manufacturer’s protocol. Finally, the immune mononuclear cell layer was collected, and cells were centrifuged and stained for flow cytometry. The remaining cell pellet was collected to analyze epithelial cells by using the epithelial markers.

### 2.8. Flow cytometry

Single cells from AOM/DSS mice colon were stained for flow cytometry. Dead cells were stained with Fixable Viability Dye (FVD), and then with different lectins, namely L-PHA and *Galanthus Nivalis* Lectin (*GNA*), which recognizes mannose residues. After that, extracellular and intracellular markers were stained: anti-CD45, anti-CD3E, anti-CD4, anti-CD8a, anti-γδTCR, anti-PD-1, anti-RORγT, anti-EpCam, anti-MCH-I, anti-PD-L1. Data acquisition was performed in the FACS Canto v.2 flow cytometer (BDBioscience), using the FACSDiva software (BDBioscience). For single cells from fresh colon biopsies, cells were stained with FVD, L-PHA, GNA, and afterward with anti-human antibodies: anti-CD45, anti-CD3, anti-CD4, anti-CD8, anti-γδTCR, and anti-IFNγ. Data acquisition was performed in the Cytek Aurora flow cytometer (Cytek), using the SpectroFlo (Cytek). The files were then analyzed with FlowJo software version 10.8.1. Antibody and lectin clones and dilutions are described in Table S3.

### 2.9. Colonoscopies

Mice were anesthetized with isoflurane and colonoscopies were performed by a veterinary doctor using the Mainz Coloview System.

### 2.10. RNA isolation and real-time PCR

RNA was isolated from mice frozen colon tissue at different timepoints (dysplasia or carcinoma) using TRI reagent (Sigma-Aldrich) according to the manufacturer’s instructions. Total RNA was quantified using Nanodrop One, and cDNA synthesis was performed using SuperScript IV Reverse Transcriptase (Invitrogen) according to the manufacturer’s protocol. Real-time PCR was performed in 96-well reaction plates, and cDNA was amplified using LightCycler 480 SYBR Green I Master (Roche) and respective primers (IDT): *Ifng*_Fw: TGGCTGTTTCTGGCTGTTACT, *Ifng*_Rv: GTTGCTGATGGCCTGATTGTC; *Il17a*_Fw: TACCTCAACCGTTCCACGTC, *Il17a*_Rv: TTCCCTCCGCATTGACACAG; *gapdh*_Fw: GAAGGTCGGTGTGAACGGAT, *gapdh*_Rw: CTCGCTCCTGGAAGATGGTG. Amplification data were acquired with 7500 Fast Real-Time PCR System (Applied Biosystems).

*Gapdh* mRNA expression was used as a housekeeping control gene.

### 2.11. Statistical analysis

All statistical analyses were performed using GraphPad Prism 9 or the statistical software SPSS version 29.0.2.0 (IBM Corp., IBM SPSS Statistics for Mac).

The prediction capacity of L-PHA levels to discriminate patients who would progress in the carcinogenic cascade was determined by plotting the ROC curves and calculating the AUC. The cutoff that revealed the best balance between sensitivity and specificity was selected for the subsequent statistical analysis. Colon sections exhibiting ≥50% L-PHA reactivity were classified as “high expression” and sections with <50% L-PHA reactivity were classified as “low expression”. Chi-square test (Fisher’s exact test) was performed to test the ability of high L-PHA and other variables (age at diagnosis, therapy, and colitis extension) in predicting progression. Moreover, multivariable logistic regression analysis was performed to determine the variables that could independently predict progression to dysplasia or cancer. For the logistic regressions, the Hosmer–Lemeshow test was performed.

Outlier and normality tests were performed for all experiments prior to analysis and identified outliers were excluded. Statistical significance was calculated by 2-tailed unpaired *t*-test or 2-way analysis of variance (Kruskal–Wallis test, 2-way ANOVA, or multiple *t*-test) when more than 2 experimental groups were compared. All data are represented as means ± SEM or with the min to max, showing all points. *P*-values are indicated as follows: *P < .05, **P < .01, ***P < 0.01, and ****P < .0001.

## 3. Results

### 3.1. Changes in *N-*glycosylation profile of immune cells occur during CAC development: predictive potential in carcinogenic progression

**Figure 1.**
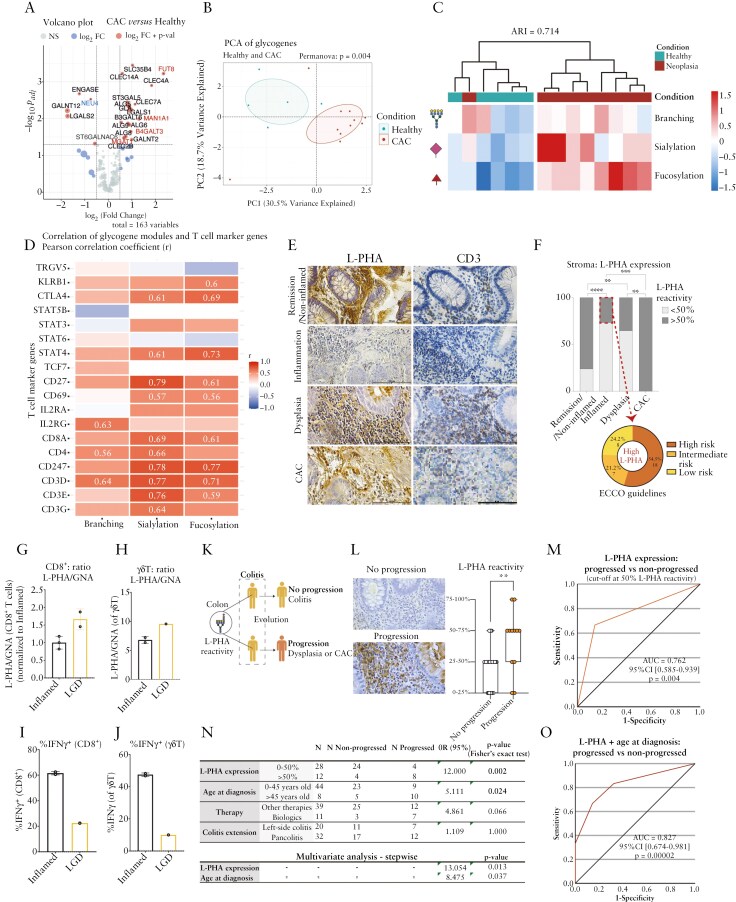
Increase in glycans complexity along CAC development impacts immune profile and predicts progression in risk patients. (A) Volcano plot of differentially expressed genes in CAC samples matched with healthy individuals, showing log_2_fold change versus −log10P (adj). Letters in red highlight genes differentially expressed involved in the *N*-glycosylation pathway. Dashed lines indicate the *x*-axis threshold of |log_2_[fold change] >0.5 and the *y*-axis threshold of log10P(adj) < 0.05. (B) Principal component analysis of *N*-glycan genes expression (*MAN2A1*, *MAN2A2*, *MAN2B1*, *MAN2B2*, *MGAT1*, *MGAT3*, *MGAT5*, *MGAT5B*, *ST3GAL1*, *ST3GAL2*, *ST3GAL4*, *ST3GAL6*, *ST6GAL1*, *FUT2*, *FUT8*) of colonic mucosa of CAC patients and healthy controls. Data are plotted as scatterplots of samples into the low-dimensional space defined by the first and second principal components (PC1 and PC2). (C) Hierarchical heatmap of glycogene modules representing unsupervised clustering between UC patients with neoplasia and healthy donors. (D) Correlation analysis between glycogene modules and genetic T-cell markers. Correlations tested between genetic markers for T cells (populations, activation, differentiation, and exhaustion) and glycogene clusters through Spearman’s method. Red and blue colors represent positive and negative correlations, respectively. Values are only shown for significant correlations (*P* < .05). (E) Representation of L-PHA and CD3 histochemistry from non-inflamed, inflamed, dysplasia, and carcinoma (CAC). Scale bar, 50 µm. (F) Qualitative classification of L-PHA reactivity in the stroma compartment along CAC cascade (non-inflamed/remission (*n* = 25), inflamed (*n* = 124), dysplasia (*n* = 34), CAC (*n* = 7)). Discrimination of high and low L-PHA reactivity from inflamed samples according to the ECCO guidelines: low risk, intermediate risk, and high risk. (G–J) L-PHA/GNA mean fluorescence intensity (MFI) ratio and IFNγ production by CD8^+^ T cells and γδT cells from human biopsies with inflammation (*n* = 2 or 3) and LGD (*n* = 1 or 2). (K) Schematic representation of assessment of risk of progression to cancer in patients under colitis. (L) Representative L-PHA histochemistry and qualitative classification of L-PHA in the stroma from inflamed tissue either from the colon zone prior to the development of dysplasia/CAC (“progressed” group) or from the left-side colon (“not progressed” group). (M) Receiver-operating characteristic (ROC) curve for L-PHA reactivity in samples from patients with IBD that progressed to dysplasia/CAC and patients that did not progress. (N) Predictive capacity of high L-PHA reactivity and other risk factors (age at diagnosis, therapy, and colitis extension) in distinguishing patients with IBD that will progress to dysplasia/cancer. (O) ROC curve plotted for the predicted probabilities of the combination of L-PHA reactivity and age at diagnosis from patients with IBD that progressed or did not progress in the cascade. 95% CI, 95% confidence interval; AUC, area under the curve; CAC, colitis-associated colorectal cancer; IBD, inflammatory bowel disease; LGD, low-grade dysplasia; L-PHA, Phaseolus vulgaris Leucoagglutinin; OR, odds ratio; UC, ulcerative colitis.

In order to access the impact of cellular glycosylation in the colon microenvironment along CAC development, we started to conduct an *in silico* analysis using a public human dataset (GSE37283) that included transcriptomic (microarray) data from healthy individuals (*n* = 5) and UC with neoplasia patients (*n* = 11). Differential gene expression analysis was performed, and the upregulation and downregulation of several glycosylation-related genes were observed in UC with neoplasia samples in comparison with healthy controls (Figure 1A, Table S4). Specifically, we observed that genes participating in the *N-*glycosylation pathway such as *MAN1A1*, *MGAT1*, *FUT8*, and *B4GALT3* were significantly upregulated in neoplasia when compared with healthy subjects (Figure 1A). Subsequently, PCA was performed for a subset of key glycogens involved in the *N*-glycosylation pathway (*MAN2A1*, *MAN2A2*, *MAN2B1*, *MAN2B2*, *MGAT1*, *MGAT2*, *MGAT3*, *MGAT5*, *MGAT5B*, *ST3GAL1*, *ST3GAL2*, *ST3GAL4*, *ST3GAL6*, *ST6GAL1*, *FUT2*, *FUT8*) revealing a distinct glycogene expression profile in neoplasia patients when compared with healthy controls (Figure 1B). Hierarchical clustering analysis revealed 2 distinct clusters corresponding to healthy versus neoplasia conditions, uniquely based on the expression patterns of glycogenes. Notably, neoplasia patients exhibited a tendency to upregulate several glycogenes, including *MGAT1*, *MAN2A1*, *MAN2B1*, and *MGAT2* (which contributed to the clustering) (Figure S1A), associated with the initial steps of the biosynthesis of more complex *N*-glycan structures. Additionally, upregulation of glycogenes related ro sialylation and fucosylation was also observed in neoplasia patients (Figure S1A). Additionally, in order to consider the contribution of the different glycogenes for the complete synthesis of complex branched *N*-glycans, sialylation, and fucosylation, we clustered the glycogenes according to their biological function in terms of the synthesis of complex branched glycans (including the *MGAT1*, *MAN2A1*, *MAN2B1*, *MGAT2*, *MGAT3*, and *MGAT5*); the synthesis of sialylated glycans (ST3GAL1 and ST6GAL1); and fucosylation (FUT2 and FUT8). A hierarchical clustering analysis was performed comparing neoplasia samples and controls (Figure 1C), and the results showed that the combination of these sets of glycogenes effectively separates healthy controls from neoplasia cases. The separation was quantitatively assessed using the ARI, showing an ARI of 0.714 which indicates a strong agreement in distinguishing the 2 groups. These findings point toward the biological relevance of *N*-glycosylation-related pathways in CAC development.

Then, we evaluated whether this differential glycogene expression detected in the colon of neoplasia patients was associated with T-cell-related genes. For that, the glycogenes associated with the *N*-glycosylation pathway (namely, *MGAT1*, *MAN2A1*, *MAN2B1*, *MGAT5*, *MGAT5B*, *ST3GAL1*, *ST6GAL1*, *FUT2*, *FUT8*) were specifically correlated with different T-cell-associated genes (such as *CD3D*, *CD3E*, *CD3G*, *CD4*, *CD8A*, *TRGV5*—markers of T-cell populations, and *CD69*, *STAT3*, *STAT4*, *STAT5B*, *STAT6*, *CTLA4*—activation-related markers) (Figure S1B, Table S5). Notably, we found a pronounced positive correlation between the expression of glycogenes from the first steps of complex *N-*glycans biosynthesis (*MGAT1*, *MAN2A1*, and *MAN2B1*) with the T-cell markers *CD3*, *CD4*, and *CD8* genes, as well as with the signaling cascade STAT4, which induces T helper 1 (Th1) differentiation (Figure S1B). Importantly, *MGAT5* glycogene, previously described to impose regulatory properties on T cells,^16,23,35^ showed an overall negative correlation across nearly all T-cell markers (even though not significant), suggesting that high levels of branched *N*-glycans are correlated with decreased T-cell activation, as previously observed.^16^ Furthermore, genes involved in sialylation (*ST3GAL1* and *ST6GAL1*) and fucosylation (*FUT2* and *FUT8*) were also correlated with genetic markers of T cells (Figure S1B). As previously described, a correlation analysis using the cluster of glycogenes participating in the synthesis of complex branched *N*-glycans, sialylalated, and fucosylated glycans (Figure 1D) was performed. The results showed that the branching module was highly correlated with the expression of the T-cell markers *CD3G*, *CD4*, and *ILR2G*.

These results support the biological relevance of the overexpression of the *N*-glycosylation pathway in CAC development, specifically in correlation with the T-cell response. Further correlation analysis between the same set of glycogenes and other immune cell markers, such as B cells, macrophages, and dendritic cells, was also performed, revealing minimal to no significant correlations (Figure S1C–E), further supporting the specific impact of the *N*-glycosylation process in T-cell response during CAC development.

In order to further validate these *in silico* results, the expression profile of complex *N-*glycan structures along CAC development was characterized in 190 FFPE colon tissue samples derived from 47 UC and 5 Crohn’s disease (CD) patients, 25 in remission (20 UC/ 5 CD), 124 with active disease (105 UC/19 CD), 34 with dysplasia (31 UC/ 3 CD), and 7 with CAC (only derived from UC). All samples were obtained from long-standing IBD patients (>8 years of diagnosis) with increased risk for neoplastic transformation (according to the ECCO guidelines^9^) from 2 different cohorts, from Portugal and Spain. The results showed a decreased expression of complex branched *N*-glycans (β1,6-GlcNAc branched *N*-glycans, detected by the lectin L-PHA) in the inflammatory infiltrate (positive to CD3) in inflamed/active colitis when compared to non-inflamed/remission colitis. The levels of expression of complex branched *N*-glycans tend to increase in the colonic inflammatory infiltrate in patients with dysplasia, reaching the highest levels of expression in patients that develop CAC (as observed by IHC and IF), indicating a possible impact during the transition from dysplasia to CAC (Figure 1E and F, Figure S2A). Interestingly, IBD patients with inflammatory activity and exhibiting higher reactivity to L-PHA (high levels of expression of branched *N*-glycans) are those displaying a higher risk of progressing to CAC (accordingly with ECCO guidelines for risk of malignancy in IBD patients) (Figure 1F). Furthermore, the analysis of a small subset of fresh colonic biopsies from long-standing IBD patients derived from inflamed (*n* = 2 or 3) and LGD tissue (*n* = 1 or 2) revealed that LGD biopsy exhibits a slight increase in the L-PHA/GNA ratio in CD8^+^ and γδ T cells in comparison with inflamed tissue (Figure 1G and H, Figure S2B and C), alongside with a decrease in the percentage of IFNγ production by both CD8^+^ and γδ T cells (Figure 1I and J, Figure S2D and E). Interestingly, when we compare fresh colonic biopsies from healthy controls with inflamed tissue, we observe a decrease in complex *N*-glycans in general CD3^+^ T cells and both CD8^+^ and γδ T cells (Figure S2F–H), corroborating the results from IHC and IF.

**Figure 2.**
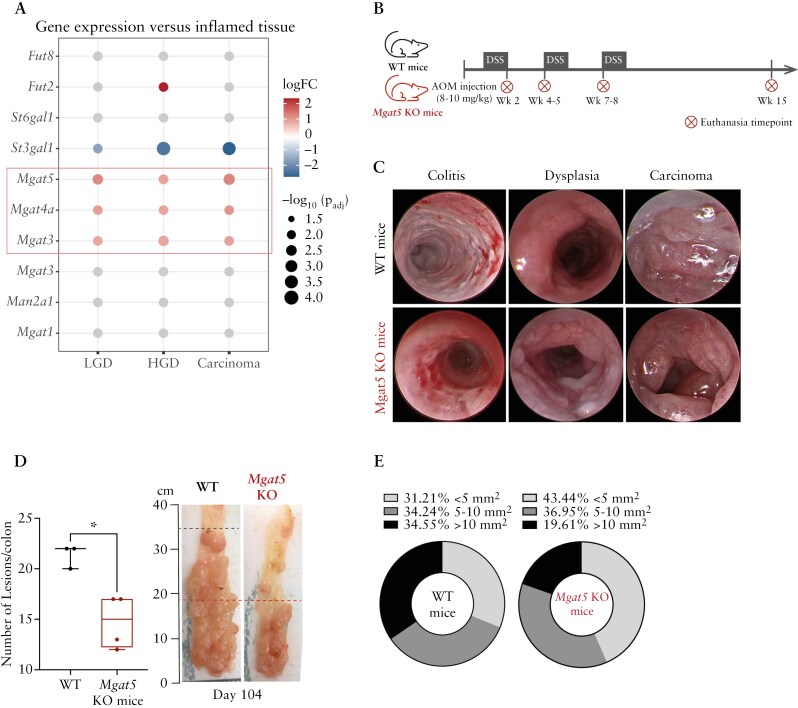
Complex branched *N*-glycans impact tumor growth and size. (A) Dot plot comparing the expression profiles of glycogenes associated with the *N*-glycosylation pathway between inflamed and subsequent stages. Red and blue colors represent positive and negative fold changes, respectively. Colored dots are only shown for significant *P*-values (*P* < .05). (B) Schematic representation of the AOM/DSS mouse model performed in WT and *Mgat5* KO mice. (C) Colonoscopies of mice colon in different disease stages (colitis, dysplasia, carcinoma), comparing WT mice with *Mgat5* KO mice. (D) Number of lesions per mice in the last timepoint (day 104) of WT (*n* = 3) and *Mgat5* KO mice (*n* = 4). Representative image of the distal colon in the last timepoint (day 104) of WT and *Mgat5* KO mice. Lines show the extension of the tumor lesions. (E) Segregation of lesions per size in the last timepoint (day 104) of WT (*n* = 3) and *Mgat5* KO mice (*n* = 4). AOM, azoxymethane; DSS, dextran sulfate sodium; KO, knockout; LGD, low-grade dysplasia; HGD, high-grade dysplasia; WT, wild type.

Finally, to gain further insight into the predictive power of the expression levels of complex branched *N*-glycans to identify colitis patients at risk for CAC, we performed an ROC analysis. Samples from IBD patients under inflammation were stratified into 2 groups: (1) patients who later progressed in the carcinogenic cascade, developing either dysplasia or CAC (*n* = 19), and (2) patients who did not progress on the cascade (*n* = 28) (Figure 1K). Inflamed samples collected prior to the carcinogenic development were selected from either the same colon zone as the dysplasia/CAC (for the “progressed” group) or from the left-side colon, the most predominant side for CAC development (for the “non-progressed” group). Tissue samples from patients who progressed to dysplasia were sampled from an average of 1.5 years (1–15 years, with a standard deviation of 4.1), prior to the development of the neoplasia. Interestingly, the results revealed that patients who have progressed in the carcinogenic cascade are those with the highest levels of L-PHA reactivity in the intestinal stroma at the colitis stage when compared to those who did not progress (Figure 1l). In addition, an ROC curve was performed to test the predictive performance of branched *N-*glycosylation levels in the stroma, at colitis stage. The cutoff value that presented the best balance between specificity and sensitivity corresponded to 50% of L-PHA reactivity. Interestingly, using the defined cutoff of 50%, our results showed that the levels of branched *N-*glycans prior to the development of dysplasia/CAC were capable of predicting the progression with 85.7% specificity and 66.7% sensitivity (AUC of 0.762) (Figure 1M). Other clinical parameters, including the age at diagnosis, the use of biologics therapy, as well as the extension of colitis, were also analyzed. ROC curve analysis of the different clinical parameters revealed that only the age at diagnosis (cutoff of 45 years old) exhibited significant predictive value in determining cancer progression, with 82.1% specificity and 52.6% sensitivity (AUC of 0.674) (Figure 2I–K). Statistical analysis demonstrated that high L-PHA reactivity increased the odds of progression by 12-fold (*P* = .002) when compared with patients with low L-PHA reactivity (Figure 1N). Besides L-PHA, the age at diagnosis also increased the odds of progression by 5-fold (*P* = .024) (Figure 1N). Importantly, the multivariable analysis revealed that L-PHA expression (13-fold) and age at diagnosis (8-fold) are capable of predicting the progression from colitis to dysplasia and/or CAC independently of the other clinical parameters (Figure 1N). Taking into account that the L-PHA expression and age at diagnosis were the only independent predictors of progression, we computed an ROC curve analysis using these 2 parameters together. The combination revealed the capacity to predict CAC progression with sensitivity of 83.3% and specificity of 67.9% with an AUC of 0.827, which revealed the increased predictive performance of both biomarkers together in defining the risk of progression to CAC (Figure 1O).

Taken together, these results revealed the biological relevance of the branched *N-*glycosylation pathway in CAC development, in association with T-cell response. In addition, we also demonstrated the predictive performance of the expression levels of complex branched *N*-glycans detected in the inflammatory microenvironment (stroma) as a tool that, in combination with age at diagnosis, displays the capacity to predict the risk of CAC progression (at colitis stage) with a promising sensitivity and specificity. Although these findings show promising results, there is a need to validate this association in a larger and prospective cohort of colitis-associated dysplasia and CRC, where differences between UC and CD patients can be further dissected.

### 3.2. Expression of complex *N*-glycans increases susceptibility to CAC development *in vivo*

Given the significance of complex branched *N*-glycans in the context of IBD and CAC, we next sought to investigate the biological effects of these structures *in vivo*, during CAC development using the AOM/DSS mouse model. This mouse model mimics human CAC in terms of the malignant transformation and progression from colitis to LGD, HGD, and invasive carcinoma through the administration of the carcinogenic agent AOM, followed by induction of inflammation through three cycles of DSS.^32,36^

**Figure 3.**
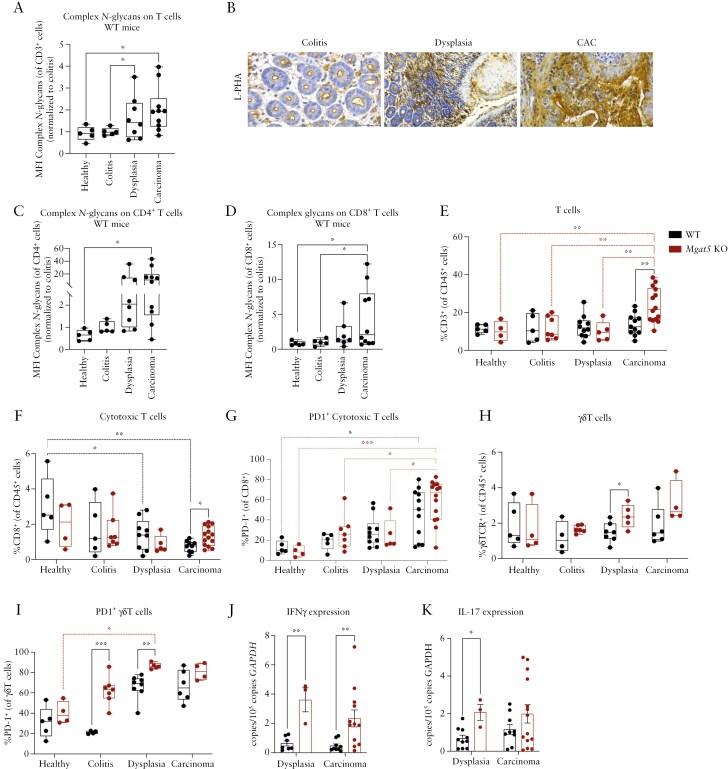
Overexpression of complex branched *N*-glycans on T cells along CAC development impacts T-cell infiltration and pro-inflammatory cytokine production. (A) Relative MFI of L-PHA staining in CD3 ^+^ T cells across tumor development. (B) Representation of L-PHA histochemistry from colitis, dysplasia, and CAC. Scale bar, 50 µm. (C, D) Relative MFI of L-PHA staining in CD4^+^ and CD8^+^ T cells across tumor development. (E) Percentage of CD3^+^ T cells in the colonic mucosa across tumor development. (F) Percentage of CD8^+^ (cytotoxic T cells) in the colonic mucosa across tumor development. (G) Percentage of PD-1^+^ cells in cytotoxic T cells (CD8^+^) populations. (H) Percentage of γδT cells in the colonic mucosa across tumor development. (I) Percentage of PD-1^+^ cells in γδT cell populations. (J, K) RT-PCR of IFNγ and IL-17 cytokines in colon samples from dysplasia and carcinoma stages. WT mice—black bars; *Mgat5* KO mice—red bars. Histological classification of samples was performed by a pathologist, WT mice: healthy (*n* = 5), colitis (*n* = 5), dysplasia (*n* = 10) and carcinoma (*n* = 11), and *Mgat5* KO mice: healthy (*n* = 5), colitis (*n* = 7), dysplasia (*n* = 5), and carcinoma (*n* = 16). Identified outliers were excluded. Statistics were obtained from the individual *P*-values of the 2-way ANOVA with multiple comparisons between the different stages. Multiple *t*-test was performed to compare the 2 groups in the different stages. *P < .05; **P < .01; and ***P < .001.

First, using a publicly available dataset (GSE31106) derived from AOM/DSS model, we performed an *in silico* analysis of a list of selected glycogenes involved in branching *N*-glycosylation (Tables S6 and S7). We observed a significant overexpression of *Mgat5* glycogene as well as *Mgat4a* and *Mgat3* in LGD, HGD, and carcinoma, when compared with inflamed tissue, suggesting an overall increase in complex *N*-glycosylation pathway along CAC development (Figure 2A), which is in accordance with our human results (Figure 1). Accordingly, a decrease in the expression of these glycogenes was observed in inflamed tissue when compared with healthy tissue (Figure 3A), which is in line with the negative correlation between branched *N*-glycans and T-cell-mediated inflammation, as previously described ^16,29^.

Afterward, using a glycoengineering mouse model, *Mgat5* KO mice, which displays a deficiency in complex β1,6-GlcNAc branched *N*-glycans (Figure S3B), we evaluated the impact of a deficiency in *Mgat5*-mediated branched *N*-glycosylation in CAC susceptibility. Thus, AOM/DSS model was performed in both *Mgat5* KO mice and WT mice (with a normal *N*-glycosylation pathway) and multiple euthanasia timepoints were defined to analyze different stages of carcinogenesis, from colitis to dysplasia and cancer (Figure 2B). The mice were monitored through colonoscopies to assess tumorigenic progression (Figure 2C). H&E staining of the colon allowed the histological evaluation of the tissue at each CAC developmental stage, assessed by a pathologist (Figure S3C). Notably, histopathological evaluation at the multiple time points of CAC progression revealed that WT mice exhibited more undifferentiated lesions with an invasive morphology prior than *Mgat5* KO mice (Figure 3C). In addition, *Mgat5* KO mice exhibited, at the latest timepoint, a significantly lower number of tumor lesions (Figure 2D) with smaller sizes (Figure 2E) than WT mice, which support that a deficiency in *Mgat5*-mediated branched *N*-glycans delays CAC progression.

Together, these findings support the biological effect of branched *N*-glycans in promoting CAC development *in vivo*, pinpointing branched *N*-glycosylation as a potential new mechanism (encoding a potential risk biomarker) underlying the transition from inflammation to cancer.

### 3.3. Overexpression of complex branched *N*-glycans on mucosa T cells along CAC development regulates cytotoxic T-cell-mediated immune response and colon cancer susceptibility

In order to gain mechanistic insights on the impact of branched *N*-glycosylation pathway in the regulation of T-cell-mediated immune response associated with CAC development, we next sought to characterize the dynamics of T-cell glycosylation profile, both CD4^+^ and CD8^+^, along the carcinogenic cascade *in vivo*. Upon exposure to the AOM/DSS model, we observed that infiltrated T cells exhibit a gradually increased expression of branched *N-*glycans along CAC development (Figure 3A and B), from inflammation to premalignancy and cancer, both on CD4^+^ and CD8^+^ T cells in WT mice (Figure 3C and D). This dynamic modification of colonic T cells, with increased levels of branched *N*-glycans along CAC development, was accompanied by an increase in tumor burden (Figure 2D). *Mgat5* KO mice with an absence of branched *N*-glycans on T cells (Figure S4A) displayed a more protective phenotype in terms of CAC development. In accordance, the absence of β1,6-GlcNAc branched *N*-glycans in *Mgat5* KO mice was balanced by an increase in mannosylated *N-*glycans (less complex type of glycans) on T cells in advanced stages of the disease (dysplasia and carcinoma) (Figure S4B). This altered T-cell glycoform observed in KO mice, with a deficiency in branched *N*-glycans and increased levels of mannose-enriched glycans, appears to confer effector functions to T cells (as described previously^16,27^) and antitumor effect as described below. Interestingly, this dynamic regulation of the glycosylation profile along CAC development appears to be specific for tumor-infiltrated T cells and not epithelial cells, as no changes in complex branched *N-*glycans were observed in colonic epithelial cells along the disease progression (Figure S4C).

Then, to further investigate how changes in T-cell glycosylation observed during CAC development affect their antitumoral activity, we conducted a comprehensive functional characterization of the T cells in the colon at each stage of CAC development. We demonstrated that the absence of β1,6-GlcNAc branched *N*-glycans in *Mgat5* KO mice was accompanied by a significantly increased infiltration of CD3^+^ T cells along CAC development when compared to WT mice (Figure 3E). This increased T-cell infiltration was both T helper (CD4^+^) (Figure S4D) and cytotoxic (CD8^+^) T cells (Figure 3F). In WT mice, where the CAC burden was higher than KO, there is a significant decrease of CD8^+^ T-cell infiltration in dysplasia and carcinoma in comparison with healthy mice, suggesting a decrease in cytotoxic T cells associated with increased tumor progression (Figure 3F). We also observed an increase in T helper 17 cells (RORγT^+^ CD4^+^ T cells) in dysplasia stage in *Mgat5* KO mice compared to WT mice (Figure S4E). Together, these results support the impact of branched *N*-glycosylation in modulating CAC development, through the regulation of T-cell-mediated immune response. Accordingly, we also demonstrated an increased expression of PD-1 in *Mgat5* KO mice T cells over time supporting the activating phenotype of these cells in KO mice (Figure 3G, Figure S4F). PD-1 molecule can also act as an immune inhibitory molecule when tumors express high levels of PD-L1 membrane receptor. Our results further showed that tumors derived from *Mgat5* KO mice presented a tendency for lower levels of the immune checkpoint inhibitor PD-L1 molecules in the carcinoma stage (Figure S4G), which is in line with the activating and non-immunosuppressed phenotype of T cells in KO mice. Moreover, *Mgat5* KO mice tumors also showed increased levels of MHC-I in both dysplasia and carcinoma compared with WT mice (Figure S4H), further supporting increased antigen presentation and antitumor immune activation. In line with this, and besides the classical antigen-dependent immune response mediated by CD8^+^T cell, we further observed that deficiency in branched *N*-glycans in *Mgat5* KO mice also stimulated increased infiltration of γδ T cells, which was significantly observed at premalignancy, namely in dysplasia stage of KO mice compared to WT mice, with a tendency to increase also in carcinoma (Figure 3H). This immune population showed an increased expression of the activation marker PD-1 both in colitis and dysplasia of *Mgat5* KO mice (Figure 3I), suggesting that the controlled tumor growth observed in KO mice is apparently due to the priming of CD8^+^ T cells alongside γδ T-cell activation that together converge to boost an effective antitumoral immune response with suppression of CAC development. This antitumoral phenotype observed in KO mice was further validated by the increased pro-inflammatory microenvironment as demonstrated by the increased expression of pro-inflammatory cytokines, including IFNγ and IL-17 in the colon tissue, showing that *Mgat5* KO mice have an increased production of IFNγ in dysplasia and carcinoma (Figure 3J), as well as an increase in IL-17A in dysplasia and a tendency in carcinoma (Figure 3K).

Altogether, these results suggest that branched *N*-glycans covering colonic T cells are dynamically regulated along CAC development, during the progression from colitis to cancer, with direct effects in the regulation of T-cell-mediated antitumoral functions associated with susceptibility to CAC development.

## 4. Discussion

One of the major clinical and therapeutic challenges in long-standing IBD patients involves the management of the risk of malignancy, with CRC being the most feared complication in chronic colonic inflammation. Although advances in the field have reported some potential biomarkers associated with risk for CAC development,^37,38^ up to now there are no reliable prognostic biomarkers able to early identify who will develop CRC. Additionally, the efficacy of endoscopic cancer screening is still poor, pointing to a gap in knowledge in the field. Previous evidence from our group revealed that levels of branched *N*-glycans are altered in mucosal T lymphocytes of active UC patients and this was associated with disease severity.^15^ Moreover, it was demonstrated that the levels of branched *N*-glycosylation in inflammatory infiltrate of IBD patients were able to predict disease severity and nonresponse to standard therapy.^39^ Accordingly, increased levels of branched *N*-glycans expressed by colorectal cancer cells were demonstrated to contribute to an immunosuppressive environment associated with tumor growth.^17^ Altogether, this evidence supporting the prominent role of branched *N*-glycans in IBD immunopathogenesis together with the fact that branched *N*-glycans also has a major role in colon carcinogenesis,^13,14,17^ prompted us to investigate the potential role of T-cell glycosylation in defining the risk for colitis-associated cancer progression. In this study, we revealed the existence of a switching event in mucosal T-cell glycosylation profile that dynamically occurs from active inflammation to dysplasia and CAC. We demonstrated the dynamic regulation of T-cell glycosylation profile, with patients with long-standing active inflammation exhibiting a gradual shift from low levels of branched *N*-glycans in T cells to a gradual increased levels of branching *N*-glycosylation in dysplasia and cancer. Importantly, from the clinical point of view, we demonstrated the potential predictive power of assessing the levels of branching *N*-glycosylation detected in the inflammatory infiltrate of colonic biopsies of patients with IBD. The combination of the levels of branched *N*-glycans together with age at diagnosis (higher than 45 years old) displayed a promising predictive performance. In fact, age at diagnosis has been associated with increased risk for cancer development,^40^ and patients older than 70 years at diagnosis had a 15-fold higher risk of developing CRC when compared with those diagnosed at younger age (<40 years).^41^ In our cohort, we showed that adults with IBD exhibiting a late diagnosis present a higher risk of progression in the carcinogenic cascade, which may be potentially explained by the long-standing, uncontrolled inflammation throughout the years. Our findings pave the way for the potential identification of a new glycobiomarker able to improve risk stratification of IBD patients for CRC development. The validation of this biomarker in larger, prospective, and multicentric cohorts and the exploitation of the combination with other clinical–pathological parameters, such as the family history of CRC, the presence of PSC), among others, are further needed.

In fact, changes in glycosylation patterns of T cells have been described to tightly regulate their activity and function both in homeostasis^24,42^ and in disease.^16,27,29,35,43^ As an example, increasing branching *N-*glycosylation on CTLA-4 molecule was shown to enhance its cell surface expression, promoting immune tolerance.^27^ Moreover, glycosylation of PD-1 has been shown to modulate its activity and response to immune checkpoint inhibitors.^44,45^ In our study, by using available microarray data from human and mouse cohorts, as well as data from our human cohort and AOM-DSS mouse model (an animal model of CAC), we demonstrated that intralesional T cells clearly exhibited a dynamic expression of glycogenes along the transition from colitis to cancer, revealing an overall promotion of the branching glycosylation machinery that regulates T-cell activity and function associated with susceptibility to CAC development and progression.

The biological relevance of the glycosylation switch on T cells associated with CAC progression was observed not only *in silico* but also in fresh colonic biopsies from long-standing IBD and CAC patients, as well as validated, at a mechanistic level, in glycoengineered mice. In fact, the deficiency of branched *N*-glycosylation, modeled in *Mgat5* KO mice, was associated with a delay in cancer development and a lower number of tumor lesions. This was closely associated with an increased infiltration of CD3^+^ T cells, both CD4^+^ and CD8^+^, in the cancer stage in comparison with WT mice. These T cells harboring a deficiency in branched *N*-glycans and increased expression of high mannose, from *Mgat5* KO mice also showed an increased activation and antitumor phenotype. This regulatory role on T cells imposed by the levels of branched *N*-glycans is in line with previous evidence showing that *Mgat5* overexpression was associated with reduced Th1 responses.^46^ In fact, the high-mannose-enriched T-cell glycome observed in *Mgat5* KO mice was already described to be associated with an increased activation of T cells and a hyperimmune response in active IBD,^16^ a glycophenotype that is lost in WT mice, fostering the transition from active inflammation, to dysplasia and cancer. Accordingly, the enhancement of branched *N*-glycans on T cells upon metabolic supplementation with glycans (GlcNAc-N-acetylglucosamine) was previously shown to suppress T-cell-mediated immune response in immune-mediated diseases,^47^ such as in IBD.^16^ Additionally, this glycan supplementation was associated with a reduction in Th1 and Th17 responses.^47^ In fact, *Mgat5* KO mice exhibited an overall increased production of potent antitumor cytokines such as IFNγ and IL-17, alongside an increased infiltration of γδ T cells that overall resulted in cancer suppression. This evidence brings to light the biological and clinical effects of deleting branched *N-*glycans as a way of promoting the activation of antitumor CD8^+^ T cells and γδ T cells, and thus in suppressing CAC development, a mechanism that deserves further exploitation envisioning a new target for CAC prevention. In fact, γδ T cells were shown to impact IBD and CAC through the production of pro-inflammatory cytokines.^48^ In our study, we observed that *Mgat5* KO mice displayed an increased expression of PD-1 in γδ T cells, which supports the relevance of glycosylation in modulating gdT-cell activity and response in CAC progression. Overall, we have here demonstrated the dynamic regulation of T-cell glycosylation profile in CAC, in which patients with long-standing active inflammation exhibit low levels of branched *N*-glycans in T cells, and during the transition to dysplasia and cancer, there is a gradual shift toward a gradual increase in branching *N*-glycosylation on T cells, with potential impact in CAC progression. While we establish the biological and clinical relevance of complex branched *N*-glycans on infiltrating T cells in CAC, this does not definitively exclude the potential role of these complex structures in other immune cells or even in epithelial cells present in the intestine.

**Figure 4.**
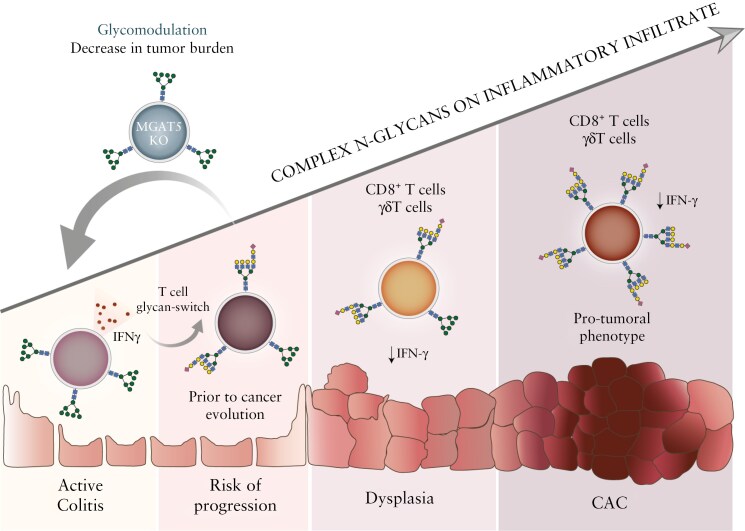
Graphical abstract. Dynamics of T-cell glycosylation as a switching event in the transition from IBD to cancer. An increase in *N*-glycans complexity in T cells occurs during the transition from inflammation to dysplasia and cancer. The dynamic increase in branching *N*-glycosylation renders T cells dysfunctional and ineffective against cancer progression during CAC development. Modulation of *N*-glycans at the surface of T cells, through the deletion of *Mgat5* gene (encoding GnT-V enzyme), imposes an effective antitumor phenotype on T cells, mainly CD8^+^ and γδT cells. The inhibition of the synthesis of complex *N*-glycans results in the increase in CD8^+^ T cells and γδT-cell frequency and activation and increased production of antitumor cytokines such as IFNγ, associated with suppression of CAC development. CAC, colitis-associated colorectal cancer; IBD, inflammatory bowel disease.

Altogether, these results, depicted in Figure 4, disclose a new cellular and molecular mechanism, based on T-cell glycosylation dynamics, that underlies the progression of long-standing IBD to CAC and reveals a potential new biomarker to improve risk stratification and cancer surveillance in IBD patients. In addition, the T-cell glycosylation shift from inflammation to cancer brings to light a putative molecular target to improve antitumoral immunity, ultimately preventing CAC development.

## Supplementary Material

jjaf043_suppl_Supplementary_Tables_S1-S7_Figures_S1-S4

## Data Availability

The data underlying this article are available in GSE37283 and GSE31106. The datasets were derived from sources in the public domain: [Gene Expression Omnibus, www.ncbi.nlm.nih.gov/geo/query/acc.cgi?acc=GSE37283, www.ncbi.nlm.nih.gov/geo/query/acc.cgi?acc=GSE31106]. All patients and mice data underlying this article are available in the article and in its supplementary material.
