# Supplementary material for: T-cell branched glycosylation as a mediator of colitis-associated colorectal cancer progression: a potential new risk biomarker in inflammatory bowel disease
Source: J Crohns Colitis. 2025 Mar 15;19(4):jjaf043. doi: 10.1093/ecco-jcc/jjaf043 (PMC12032605; doi:10.1093/ecco-jcc/jjaf043)
Supplement: jjaf043_suppl_Supplementary_Tables_S1-S7_Figures_S1-S4 [file jjaf043_suppl_supplementary_tables_s1-s7_figures_s1-s4.zip › ECCOJC_jjaf043_Suppl_Figure 1-4-Table_1-7/Leite-Gomes2025_Suppl.Figures_and_tables_edit.docx]

**Supplementary Figure 1 – (A)** Hierarchical heatmap representing unsupervised clustering between UC patients with neoplasia and healthy donors. **(B)** Correlation analysis between glycogenes and genetic T cell markers. Correlations tested between genetic markers for T cells (populations, activation, differentiation and exhaustion) and *N*-glycan complexity-related genes through the Spearman’s method. Red and blue color represents positive and negative correlations, respectively. Values are only shown for significant correlations (p < 0.05). **(C-E)** Correlation analysis between *N*-glycan complexity-related genes and genetic B cell, macrophage and dendritic cell markers. Correlations tested using Spearman’s method. Values are only shown for significant correlations (p < 0.05).

**Supplementary Figure 2 – (A)** Representative images of immunofluorescence staining of L-PHA (Green), CD3 (Red), Dapi (Blue), autofluorescence (Black) from remission/non-inflamed, inflamed, dysplasia and carcinoma (CAC). Scale bar 20 µm. On the left, slide snapshot of the selected region. Arrows point to the sites of overlap. **(B-C)** Representative histogram graphic of L-PHA and GNA staining in CD8^+^ T cells and γδT cells from human biopsies with inflammation (grey) and LGD (orange). **(D-E)** Contour plot of IFNγ-expressing CD8^+^ T cells and γδT cells from human biopsies with inflammation (n=2 or 3) and LGD (n=1 or 2). **(F-H)** L-PHA mean fluorescence intensity (MFI) in CD3^+^ cells, CD8^+^ T cells and γδT cells from human biopsies derived from healthy controls (n=3) and inflammation (n=5). **(I-K)** Receiver operating characteristic (ROC) curve for age at diagnosis (cut-off of 45 years old), therapy (use of biologics) and disease extension (pancolitis or left-side colitis) in samples from patients with IBD that progressed to dysplasia/CAC and patients that did not progress.

**Supplementary Figure 3 – (A)** Dot plot comparing the expression profiles of glycogenes associated with the *N*-glycosylation pathway between healthy colon tissue and subsequent stages (inflamed, LGD, HGD and carcinoma). Red and blue color represents positive and negative fold change, respectively. Colored dots are only shown for significant p values (p < 0.05). **(B)** Schematic image of the *N*-glycosylation pathway for the synthesis of complex branched glycans. **(C)** Histopathological evaluation by hematoxylin and eosin (H&E) staining of the WT mice and *Mgat5* KO mice during CAC mouse model (colitis, dysplasia, carcinoma). Top panel: ×40 original magnification; bottom panel: ×200 original magnification.

**Supplementary Figure 4 – (A)** Levels of complex *N*-glycans in colonic T cells (CD45^+^ CD3^+^) from WT mice and *Mgat5* KO along CAC mouse model. L-PHA lectin was used to detect complex branched *N*-glycans. **(B)** Levels of high mannose *N*-glycans in colonic T cells (CD45^+^ CD3^+^) from WT mice and *Mgat5* KO along CAC mouse model. GNA lectin was used to detect mannosylated *N*-glycans. **(C)** Levels of complex *N*-glycans in colonic epithelial cells (CD45^-^ EpCAM^+^) from WT mice along CAC mouse model. Median fluorescence intensity (MFI) was determined by flow cytometry. MFI was normalized for the average of MFI in colitis stage for the different experiments. (**D)** Percentage of CD4^+^ (T helper cells) in the colonic mucosa across tumor development. **(E)** Frequency of T helper 17 cells (RORγT^+^) in WT mice and *Mgat5* KO across the carcinogenic cascade. **(F)** Frequency of PD-1^+^ cells of T helper cells (CD4^+^) in WT and *Mgat5* KO mice across the carcinogenic cascade. **(G)** Frequency of PD-L1^+^ cells of colonic non-immune cells (CD45^-^) from WT and *Mgat5* KO mice along CAC mouse model. **(H)** Frequency of MHC-I^+^ cells of colonic non-immune cells (CD45^-^) from WT and *Mgat5* KO mice along CAC mouse model.

**Supplementary table legends**

**Supplementary table 1 –** Patients information and FFPE details regarding location, activity and risk.

**Supplementary table 2 –** Frequencies of patient details regarding disease (UC/CD), gender, age at diagnosis, therapy, presence of PSC and disease extension.

**Supplementary table 3 -** Monoclonal anti-mouse antibodies and lectins used in flow cytometry.

**Supplementary table 4 -** Differential glycogene expression profile, revealing upregulated and downregulated glycosylation-related genes in CAC human samples compared with healthy controls. Table used for the volcano plot graph.

**Supplementary table 5 –** Correlations between glycogenes and several T cell markers using a human dataset of CAC.

**Supplementary table 6 –** Glycogenes expression profile associated with the *N*-glycosylation pathway between inflamed mouse colon and subsequent stages (LGD, HGD, carcinoma).

**Supplementary table 7 –** Glycogenes expression profile associated with the *N*-glycosylation pathway between healthy colon tissue and disease stages (colitis, LGD, HGD, carcinoma).
