# Supplementary figures and images for: T-cell branched glycosylation as a mediator of colitis-associated colorectal cancer progression: a potential new risk biomarker in inflammatory bowel disease
Source: J Crohns Colitis. 2025 Mar 15;19(4):jjaf043. doi: 10.1093/ecco-jcc/jjaf043 (PMC12032605; doi:10.1093/ecco-jcc/jjaf043)

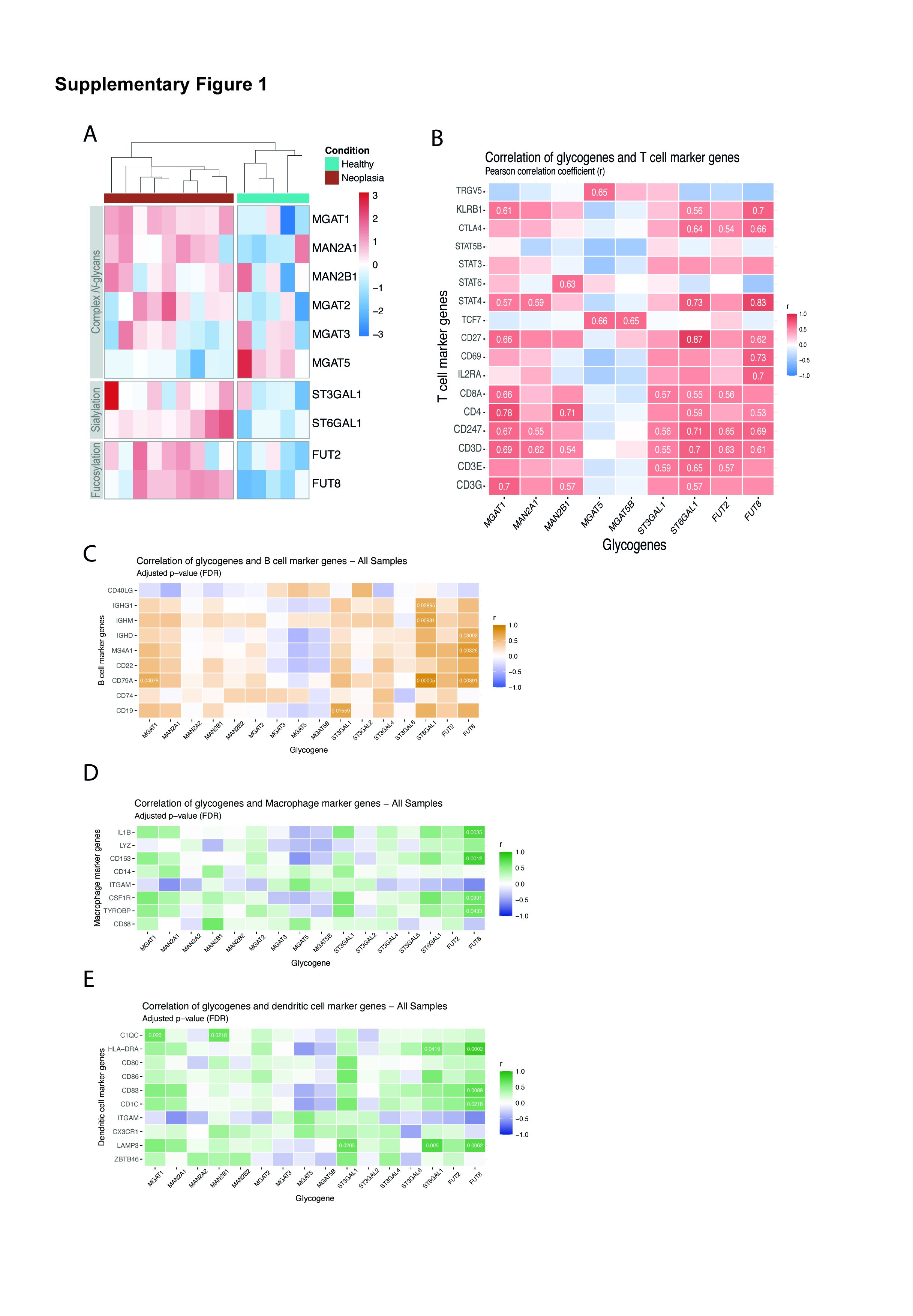

Supplement: jjaf043_suppl_Supplementary_Tables_S1-S7_Figures_S1-S4 [file jjaf043_suppl_supplementary_tables_s1-s7_figures_s1-s4.zip › ECCOJC_jjaf043_Suppl_Figure 1-4-Table_1-7/Supplementary Fig 1.tif]

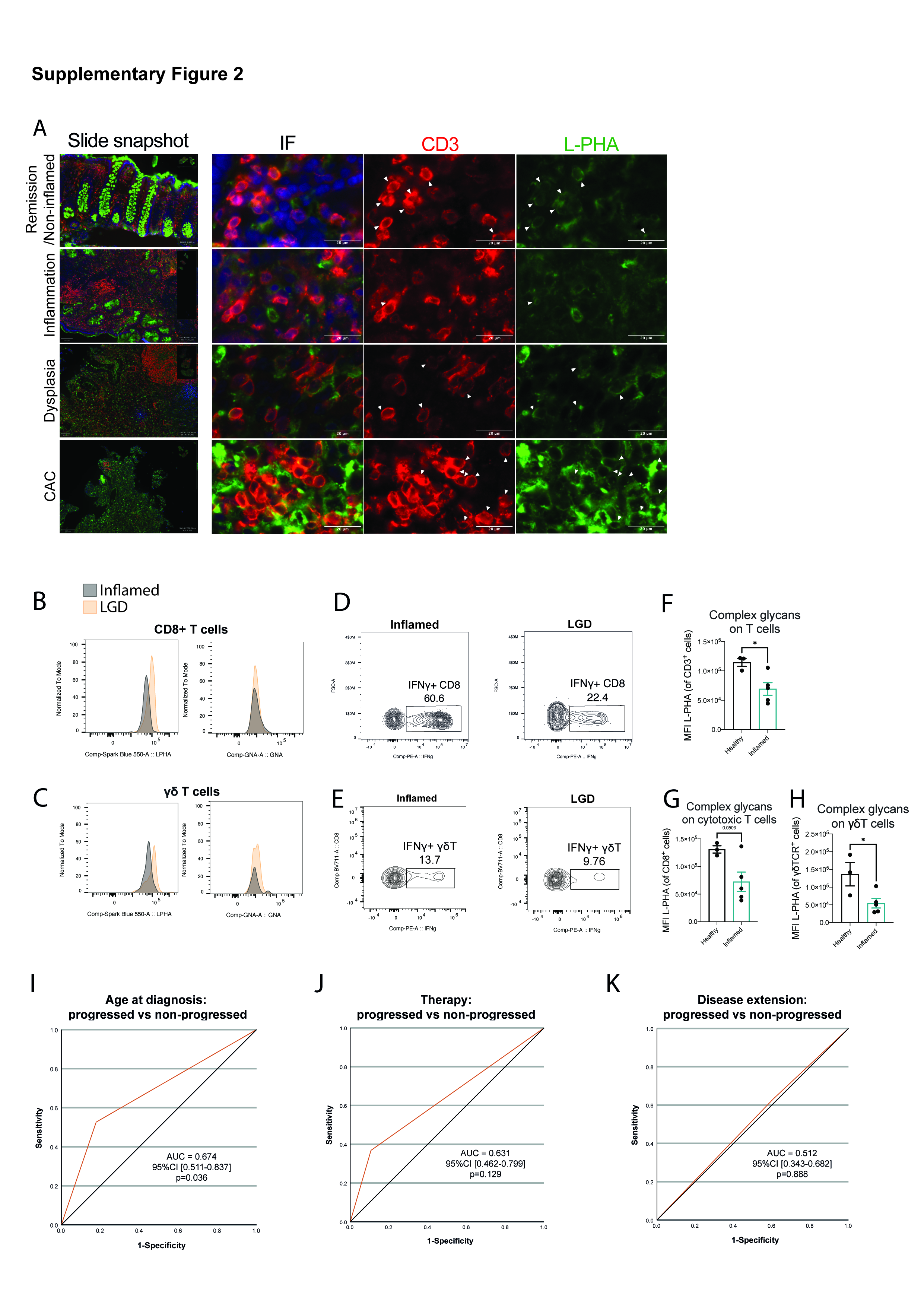

Supplement: jjaf043_suppl_Supplementary_Tables_S1-S7_Figures_S1-S4 [file jjaf043_suppl_supplementary_tables_s1-s7_figures_s1-s4.zip › ECCOJC_jjaf043_Suppl_Figure 1-4-Table_1-7/Supplementary Fig 2.tif]

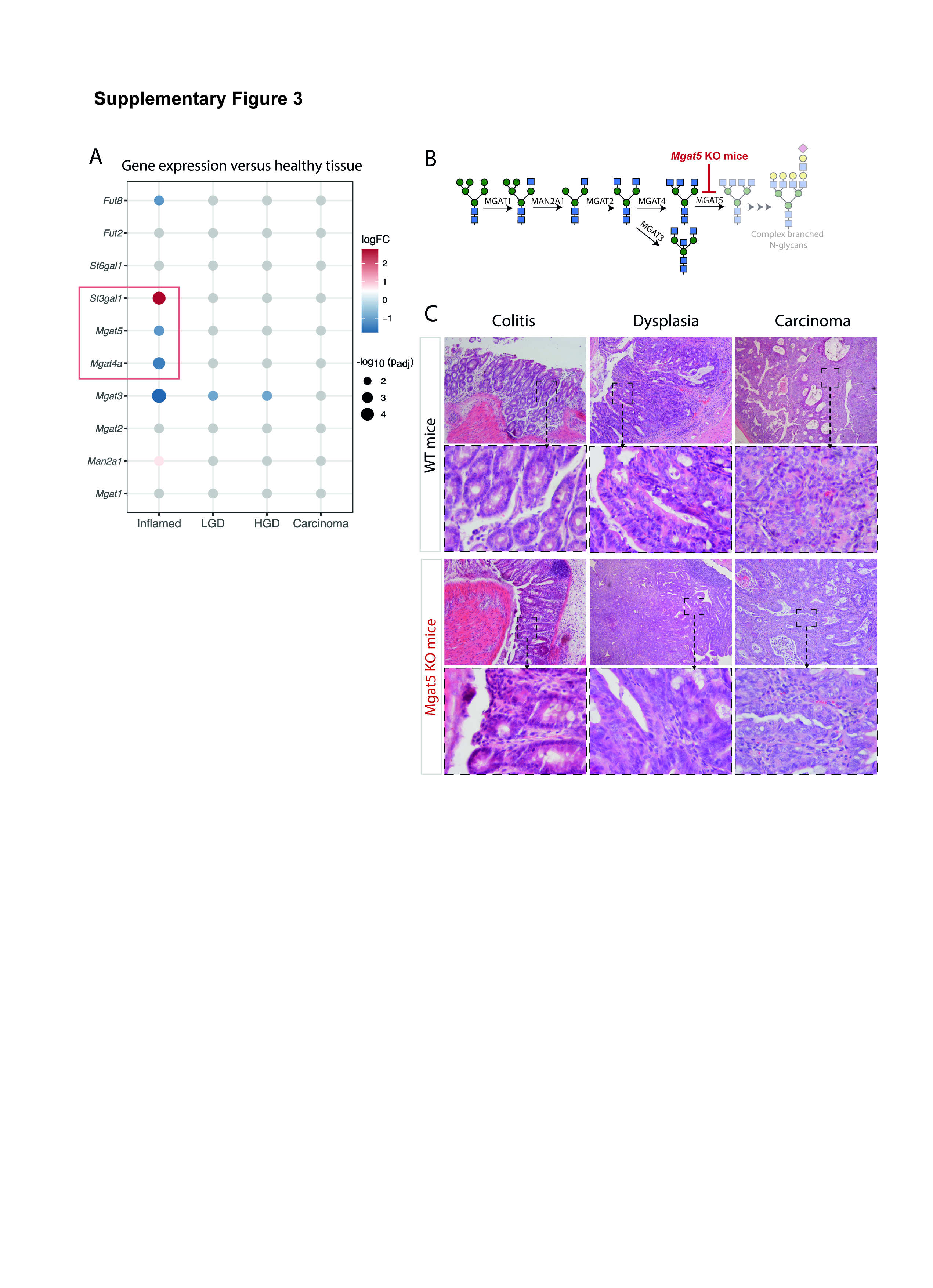

Supplement: jjaf043_suppl_Supplementary_Tables_S1-S7_Figures_S1-S4 [file jjaf043_suppl_supplementary_tables_s1-s7_figures_s1-s4.zip › ECCOJC_jjaf043_Suppl_Figure 1-4-Table_1-7/Supplementary Fig 3.tif]

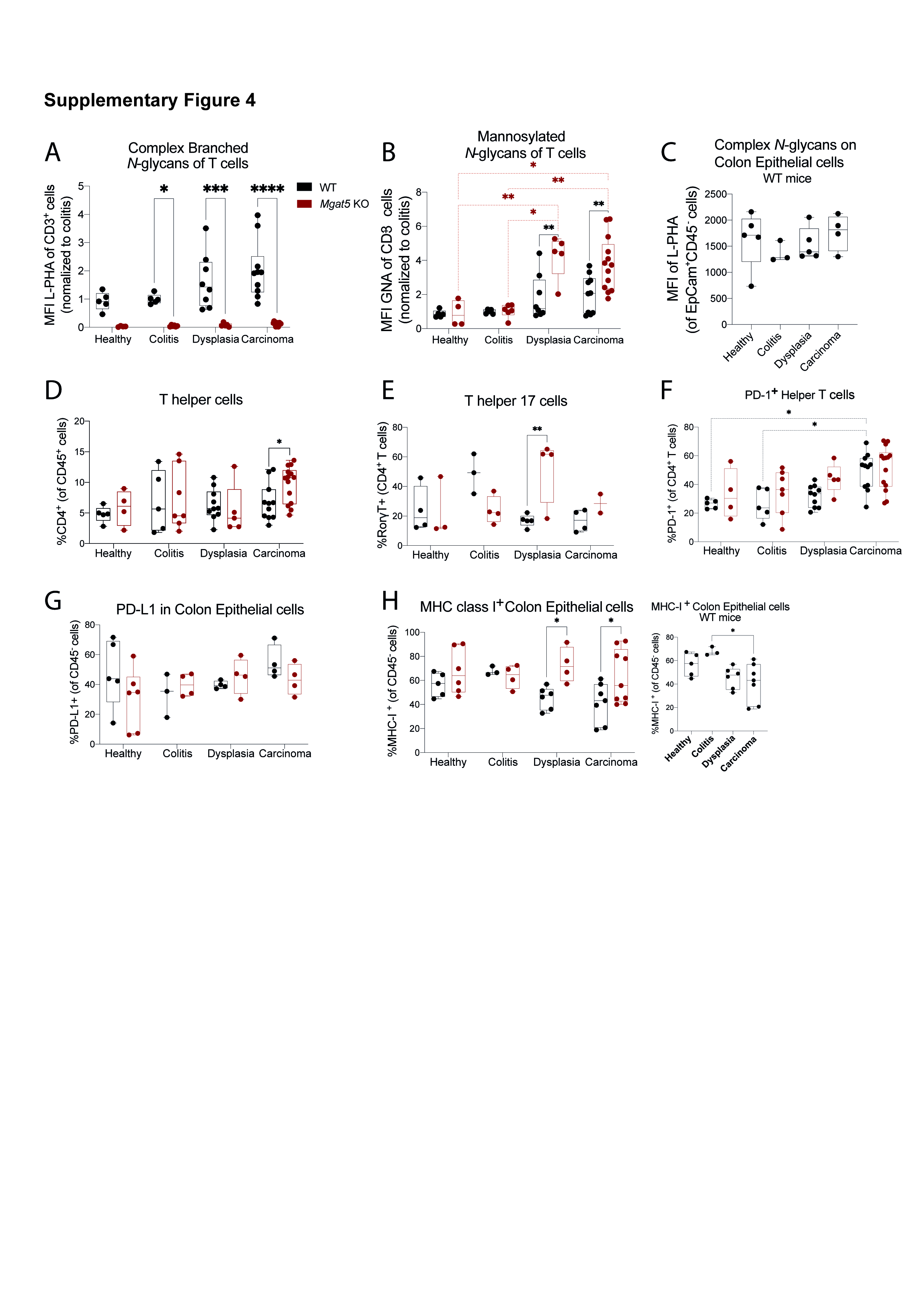

Supplement: jjaf043_suppl_Supplementary_Tables_S1-S7_Figures_S1-S4 [file jjaf043_suppl_supplementary_tables_s1-s7_figures_s1-s4.zip › ECCOJC_jjaf043_Suppl_Figure 1-4-Table_1-7/Supplementary Fig 4.tif]
